# Developing Resilience During the COVID-19 Pandemic: Yoga and Mindfulness for the Well-Being of Student Musicians in Spain

**DOI:** 10.3389/fpsyg.2021.642992

**Published:** 2021-04-21

**Authors:** L. Javier Bartos, María J. Funes, Marc Ouellet, M. Pilar Posadas, Chris Krägeloh

**Affiliations:** ^1^Department of Psychology and Neuroscience, Auckland University of Technology, Auckland, New Zealand; ^2^Department of Experimental Psychology, University of Granada, Granada, Spain; ^3^Department of Pedagogy and Singing, Royal Conservatory of Music Victoria Eugenia, Granada, Spain; ^4^Camilo Jose Cela University, Madrid, Spain

**Keywords:** yoga, mindfulness, emotional intelligence, physical activity, lockdown, higher education student musicians

## Abstract

Here, we report on a quasi-experimental study to explore the applicability and perceived benefits of the CRAFT program, which is based on mindfulness, yoga, positive psychology, and emotional intelligence, to improve higher education student musicians’ health and well-being during the lockdown. A subset of student musicians at a Higher Conservatory of Music in Spain followed the CRAFT program during the academic year 2019/2020, 1 h per week as part of their curriculum. Students enrolled in CRAFT-based elective subjects formed the CRAFT program group (*n* = 40), while other students represented the control group (*n* = 53). The onset of the national lockdown elicited by the COVID-19 pandemic occurred halfway through the program, which was subsequently delivered in an online format. We administered an online survey to explore the effect that the exposure to the CRAFT program had in terms of how participants dealt with various health and well-being concerns arising from the COVID-19 lockdown. There was a significantly higher proportion of proactive participants in the CRAFT program group, 92%, than in the control group, 58%, in terms of implementing practices to improve their health and well-being during the lockdown. Additionally, significantly more participants acknowledged perceived benefits from their practices in the CRAFT program group, 78%, than in the control group, 52%. Among proactive participants, yoga/meditation was the most implemented in the CRAFT program group, followed by exercise, and other yoga/meditation practices, whereas in the control group, exercise and Alexander technique-based practices were the most applied. In the CRAFT program group, the highest rate of perceived benefits was from yoga/meditation CRAFT-based practices, 51%, followed by exercise, 32%, and other yoga/meditation practices, 27%, whereas in the control group, benefits were reported by 29% of exercising participants and 16% for those having practiced the Alexander technique. A similar pattern was observed when excluding participants with previous yoga/meditation experience. This study revealed how participants can independently apply learned skills from the CRAFT program in response to a naturally occurring life event of unprecedented global impact, suggesting that previous exposure to mindfulness and yoga is likely to have a beneficial effect on how young adults react towards exceptionally stressful conditions.

## Introduction

The COVID-19 pandemic has had a detrimental global impact on the health and well-being of a wide range of populations beyond the elderly and other vulnerable people (Aristovnik et al., 2020; Goldman, 2020). Apart from the direct effects due to the viral infection, the lockdown implemented to counter the spread of the disease and the economic crisis triggered by it has also generated further concerns such as decreased physical activity (Ammar et al., 2020), sleep disturbances (Altena et al., 2020; Gualano et al., 2020), and psychological distress (Ozamiz-Etxebarria et al., 2020; Rajkumar, 2020; Rodríguez-Rey et al., 2020; Vicario-Merino and Muñoz-Agustín, 2020; Wang et al., 2020).

In this milieu, it appears that the prevalence of the aforementioned health and well-being concerns has also spread further among young adults undertaking tertiary education worldwide, a type of population that, prior to the onset of the pandemic, already had well-documented issues related to both psychological distresses (Bayram and Bilgel, 2008; Hunt and Eisenberg, 2010; Auerbach et al., 2016; [Bibr B10][Bibr B10] and physical problems (Hussain et al., 2013). For instance, significant reductions in physical activity of up to 49% coupled with significant increases in sedentary behaviors have been reported among Italian undergraduate students during the lockdown (Gallè et al., 2020a, b). Moreover, worsening of various conditions such as stress, depression, anxiety, negative affect, sedentarism, loneliness, sleep quality, and concerns about family and finances have been found among Greek, Chinese, Swiss, and American university students, either as compared to previous normative data (Kaparounaki et al., 2020; Liu et al., 2020), prior longitudinal data between both the same and different student cohorts (Elmer et al., 2020; Huckins et al., 2020), or even longitudinally (Li et al., 2020). In Spain, a survey completed by 3707 respondents, among which 77% were university students, revealed that 21, 28, and 34% of the sample reported moderate to extremely severe symptoms of anxiety, stress, and depression, respectively (Odriozola-González et al., 2020). Interestingly, the authors found that the highest prevalence of moderate to severe symptoms was scored by students involved in Arts and Humanities studies, a trend that has been previously documented (Lipson et al., 2016) for this discipline to which the sample of our current study – higher education student musicians – belongs.

Under these circumstances, it is reasonable to assume that for higher education student musicians who were already affected by mental and physical disturbances, this unprecedented event and its multifactorial consequences might have aggravated such conditions, negatively impacting, in turn, their lives and academic performance. As it happened with most higher education providers around the world, the onset of the lockdown disrupted the function, management, and structure of all higher conservatories of music in Spain, which, following the Spanish Government declaration of the state of emergency on March 14, started shifting into what has been called emergency remote education (Bozkurt et al., 2020). This adaptation to an online-teaching-and-learning process has added extra challenges and difficulties, overburdening the psychological and emotional pressure of both teachers – who were forced to manage dual parenting-teaching roles – and students. Therefore, if before the pandemic there was already a clear need of designing specific interventions to support higher education students’ health and well-being, the emergence of the COVID-19 and its association with the exacerbation of their pre-existing physical and psychosocial issues signified an even clearer rationale for developing such interventions. In line with this, a further inquiry regarding this claim would pose the question as to whether pre-existing health and well-being programs disrupted by the pandemic, yet tailored to be delivered online, have been effective in helping higher education students cope with the severe ensuing demands elicited by the lockdown. The current study partially addresses this research question in the context of higher education student musicians following a health and well-being program – *The CRAFT program* – as part of their curriculum at a Royal Conservatory of Music in the midst of a global pandemic and state of emergency remote education.

The recently developed CRAFT program was specifically devised to promote a holistic education that concurrently considers students’ emotional, cognitive, and physical processes to facilitate their learning experience, happiness, health, and well-being (Posadas, 2019). Though applicable to any educational context, the program was originally designed to meet the needs of students involved in specialized higher educational fields such as music, fine arts, languages, and sports. Since its creation in 2016, the program has been implemented in various educational settings and populations, with higher education student musicians at higher conservatories of music being the predominant targets. The program was first applied at the Royal Conservatory of Music of Granada Victoria Eugenia in its preliminary version called *the CRAFT’s 7 mindful minutes* in the subjects English for Musicians and Chamber Music during the academic year 2016/2017 (Rull et al., 2019; Posadas and Bartos, under review). In 2017/2018, the program was implemented as part of the academic coursework at the same institution as a CRAFT-based elective subject of Mindfulness, and the following year, the CRAFT-based elective subject of Emotional Intelligence was also added to the curriculum. The program springs from the theories, philosophical underpinnings, practices, and state-of-the-art neuroscientific findings of yoga, mindfulness, positive psychology, and emotional intelligence. These four disciplines constitute the four foundations of the program and inspired, in turn, the creation of the acronym *CRAFT*, which stands for the following Spanish terms that synthesize the five elements of the program: *Consciencia* (i.e., consciousness), *Relajación* (i.e., relaxation), *Atención* (i.e., attention), *Felicidad* (i.e., happiness), and *Transcendencia* (i.e., transcendence). Therefore, both the four foundations and the five elements represent the framework guiding the practical and theoretical content delivered as part of any syllabus designed for its implementation. In the following paragraphs, we will briefly review some of the latest findings relative to the four foundations of the CRAFT program and their potential beneficial effect for assisting higher education students to cope with the various health and well-being challenges elicited by the lockdown.

There is a growing body of research supporting the effectiveness of mind–body therapies such as yoga and mindfulness to improve various physical, mental, and emotional health-related concerns among higher education students. Not only might yoga-based practices (e.g., yoga poses, breathing, and relaxation techniques) counterbalance the stress response by vagal nerve stimulation and consequent reduction of cortisol and pro-inflammatory cytokines, but they could also enhance markers of psychophysiological health (e.g., blood pressure, heart rate, flexibility, balance, strength, pulmonary functions, and musculoskeletal conditions; Raub, 2002; McCall, 2013; Sullivan et al., 2018a). Moreover, yoga and mindfulness meditative practices might improve cognitive and emotional self-regulation and awareness by encouraging selective intentional attention, a non-judgmental attitude, and a decentering process that might reduce negative reappraisal, emotional reactivity, and rumination (Gard et al., 2014; Tang et al., 2015; Guendelman et al., 2017). In support of the above-hypothesized benefits and underlying mechanisms, researchers investigating the influence of yoga and mindfulness-based interventions on the health and well-being of university students have shown reductions in stress, depression, and anxiety (Smith et al., 2011; Falsafi, 2016; McConville et al., 2017; Breedvelt et al., 2019; Halladay et al., 2019); increased awareness, attention, memory, executive function, neuroplasticity, and overall cognitive and emotional functioning and self-regulation (Tang et al., 2007, 2010, 2015; Zeidan et al., 2010; Gothe and McAuley, 2015; Brunner et al., 2017); and the alleviation of specific concerns related to higher education student musicians such as music performance anxiety (Chang et al., 2003; Stern et al., 2012; Khalsa et al., 2013). In addition, interventions incorporating hatha yoga have shown effectiveness among higher education students in ameliorating physical abilities such as flexibility, strength, and balance (Tran et al., 2001; Park et al., 2017), and cardiovascular (Ray et al., 2001; Tran et al., 2001; Smith et al., 2011) and pulmonary functions (Birkel and Edgren, 2000; Godoy et al., 2006). Beyond these health and well-being benefits, the ancient spiritual and philosophical foundations of both yoga and mindfulness might provide higher education students with a useful asset for coping in the face of a pandemic. An explanation of how such principles can be applied to real-life situations to alleviate suffering can be found in the yoga model for self-regulation and resilience proposed by Sullivan et al. (2018b). The authors highlighted the importance of developing discrimination between *prakriti* (i.e., changing reality) and *purusha* (i.e., everlasting reality) by cultivating the witness attitude that brings the practitioner closer to *purusha* and the real self. Through this attitude, students might become more aware of their maladaptive behaviors and nurture their resilience by dis-identifying and creating distance from their *prakriti*-related concerns. A similar procedure equally derived from the experience and understanding of higher spiritual processes and pursuits is the mindful metacognitive mechanism of “reperceiving” (Shapiro et al., 2006). The potential relevance of the spiritual dimension amidst the COVID-19 pandemic has been recently underlined in a recent Spanish survey completed by 3480 participants, whereby the highest protective factor in predictive models for depression, anxiety, and stress-related symptoms was spiritual well-being (González-Sanguino et al., 2020).

In addition to yoga and mindfulness, the field of positive psychology and literature on emotional intelligence bring also valuable tools and practices to cope with the lockdown demands and difficulties. Positive psychology is majorly concerned with what goes right in people’s lives and thus focuses on cultivating positive values such as happiness, well-being, hope, gratitude, and developing one’s strengths such as courage, honesty, and resilience (Peterson and Seligman, 2004; Peterson, 2006). Positive psychology interventions encompass a series of practices based on components such as savoring, gratitude, kindness, empathy, optimism, strengths, and meaning. The cultivation of these attitudes and values might have an essential role in assisting with the various challenges elicited by the pandemic and the corresponding lockdown situation. Positive psychology interventions including components of gratitude and strengths have resulted in an increase of happiness, well-being, and decrease of depressive symptoms (Sin and Lyubomirsky, 2009). For instance, positive psychology interventions instructing participants to list grateful things per day have led to improvements in positive affect (Martínez-Martí et al., 2010), life satisfaction (Manthey et al., 2016), happiness (Mongrain and Anselmo-Matthews, 2012), and stress (Kerr et al., 2015). In addition, savoring, or the ability to be engaged in the appreciation of one’s activities and experiences, has been found to increase resilience and happiness and reduce depressive symptoms (Smith and Hanni, 2019). Lastly, knowledge about emotional intelligence provides an additional tool to face the emotional and psychological consequences of the lockdown with compassion, empathy, and care in the build-up of intrapersonal and interpersonal intelligence. Emotional intelligence places a major emphasis on monitoring one’s emotions and those of others to employ this output efficiently to support one’s behaviors effectively (Salovey and Mayer, 1990). To that end, the employment of Goleman’s (1998) five components of emotional intelligence – self-awareness, self-regulation, motivation, empathy, and social skills – might play a crucial role to offset the emotional outburst experienced by higher education students due to the lockdown. There is evidence supporting the efficacy of emotional intelligence for the promotion of positive emotions, life satisfaction, happiness, stress reduction, and well-being (Mayer et al., 2008; Ruiz-Aranda et al., 2014).

As can be drawn from this review of literature, the four foundations of the CRAFT program prescribe a series of practices and components that have been found effective for bestowing a variety of physical, mental, emotional, and spiritual health and well-being benefits among their practitioners. Therefore, a careful selection of these practices and components, tailored to suit the specific needs of higher education students within the framework of a single program, could provide them with a more complete and valuable set of coping strategies than each of these four foundations could offer alone to concurrently address their various lockdown demands and difficulties independently. The rationale supporting this supposition is based on the premise that a potential limitation of a given foundation could be strengthened by another. For instance, the theories and methods underlying both positive psychology and the research field of emotional intelligence do not make direct references to specific physical practices for health and well-being purposes, and those of mindfulness-based interventions such as the modern Mindfulness-Based Stress Reduction program (MBSR; Kabat-Zinn, 1990) include only a few postural elements drawn from *hatha* yoga. By contrast, a myriad of physical practices have been prescribed in the various styles of *hatha* yoga that sprang from the ancient yoga treatises, thus supporting the greater versatility of yoga to potentially induce physical changes and address musculoskeletal concerns than the other three foundations of the CRAFT program. In addition, though all four CRAFT foundations could offer a distinct way for developing emotional self-regulation-related skills, mindfulness and emotional intelligence components and practices place a direct emphasis and focus to train these abilities. Further details and supporting evidence about the particular practices and components drawn from, adapted from, or inspired by each foundation as part of the CRAFT program can be consulted elsewhere (Posadas, 2019; Posadas and Bartos, under review). Along these notions, a primary objective of the CRAFT program is to raise students’ consciousness of their inner responsibility to be actively engaged in their health and well-being processes, encouraging the application of the different learned techniques to real-life situations with full awareness. This is a path that places a major emphasis on the self – far away from selfishness but rather for the common benefit of developing more conscious communities – through the promotion of a self-inquiry, self-knowledge, self-responsibility, self-caring, and autonomous problem-solving attitude, whereby self-engagement stands as the driving common ground. Such a self-rooted paradigm also finds expression in the four foundations of the CRAFT program, being highly integrated into the philosophy of yoga and mindfulness-based practices. For instance, Desikachar et al. (2005) explained how yoga drives their practitioners to develop self-caring, self-responsibility, and self-inquiry health and well-being attitudes and demeanors by actively self-engaging in the different practices of yoga with a conscious disposition for finding the root of their health and well-being concerns.

The purpose of the current study was to explore the applicability and potential perceived benefits of the CRAFT program, administered during the lockdown at a higher conservatory of music, to improve student musicians’ health and well-being in comparison to a control group. Based on the abovementioned evidence supporting the effectiveness of the four foundations of the CRAFT program for improving a variety of health and well-being concerns and their emphasis on nurturing self-promoting attitudes and behaviors, we postulated the following hypotheses: The first hypothesis was that there would be a higher proportion of participants in a CRAFT program group (i.e., participants enrolled in CRAFT-based elective subjects) than in a control group (i.e., participants enrolled in other elective subjects) that would be proactive in terms of implementing practices as coping strategies to improve their health and well-being during the lockdown. This may be because participants in a CRAFT program group might have developed a higher level of self-engagement for taking care of their own health independently than participants in a control group. Subsequently, we also expected that among proactive participants in a CRAFT program group, there would be a higher proportion of participants implementing CRAFT-based practices than other types of practices as coping strategies to improve their health and well-being during the lockdown. The second hypothesis was that the proportion of proactive participants perceiving benefits from their practices implemented would be higher in a CRAFT program group than in a control group. This may be because participants in the CRAFT program group may have acquired new skills and learned health practices that may have provided them with an early foundation that they can now draw on effectively during stressful times brought about by the pandemic. Subsequently, we also expected that among proactive participants in a CRAFT program group, there would be a higher proportion of participants perceiving benefits from CRAFT-based practice implementation than from other types of practices.

## Materials and Methods

### Study Design, Setting, Participants, and Procedures

The present study was designed in response to a naturally occurring life event of unprecedented dimensions within the context of an ongoing longitudinal investigation, which is still in progress and will be reported elsewhere. As such, various aspects of the present study derived from the pre-existing longitudinal design and the unexpected emergence of the COVID-19 pandemic. Below, we describe this procedure in detail within its pertinent context and timeline.

#### Current Study Context: Longitudinal Study

Each year since the curricular application of the CRAFT program in 2017 at the Royal Conservatory of Music of Granada Victoria Eugenia, a longitudinal research project, approved and funded by the Regional Government of Andalusia, was conducted to examine the influence of the program on student musicians’ physical, cognitive, and psychological abilities, health, and well-being. In the academic year 2019/2020, the application of the program in the CRAFT-based elective subjects of Mindfulness and Emotional Intelligence started on November 6, 2019. As in all elective subjects, instruction occurred once a week for 1 h over the course of the entire teaching phase of the academic year that ended on May 13, 2020. After obtaining ethical approval from the Institutional Review Board of the University of Granada (n° 1009/CEIH/2019), the third longitudinal study was advertised to the students by the conservatory’s website, fliers, and announcements given by different faculty members. From January 13–29, 2020, student musicians were recruited through brief presentations of the study objectives and requirements held at the music conservatory. It was during these presentations that prospective participants were informed that their participation was voluntary, anonymous, and not linked to the marks of any of their subjects, and that they could withdraw from the investigation at any time and with no consequences for their studies. Therein, 197 interested student musicians signed a consent form and completed most of the baseline assessments. In order to safeguard their anonymity, they were instructed to assign themselves an alphanumeric code, to be provided in all tests and surveys related to the study. In the same recruitment session, students were informed of the possibility to enroll in an extracurricular workshop related to the investigation in exchange for credits towards their studies.

Group allocation was based on student musicians’ enrolment in pre-existing elective subjects. As a result, participants enrolled in the CRAFT-based elective subjects of Mindfulness and Emotional intelligence formed the CRAFT program group (*n* = 82), whereas participants enrolled in elective subjects, not including the CRAFT-based ones such as history of Spanish music, ergonomics, ethnomusicology, ensemble, foundations of direction, German, and English, served as a control group (*n* = 115). The intervention designed for the longitudinal study continued as planned until the emergence of the COVID-19 pandemic and the commencement of the national lockdown.

#### The Emergence of COVID-19 Gives Rise to the Current Study

The onset of the lockdown elicited by the COVID-19 pandemic occurred on March 14, 2020, halfway through the longitudinal study, a time at which the program had already been running for 16 weeks. As a result, both the program and the study were adapted to continue in an online format for the ensuing weeks. At this crucial stage, we considered that the particular implications of such an unprecedented event relative to student musicians’ participation in a pre-existing health and well-being program were worthy of investigation as a separate study. This led us to formulate a new set of hypotheses, driving the design and purposes of the current study reported herein, to explore the applicability and potential perceived benefits of the CRAFT program to improve student musicians’ health and well-being during the lockdown in comparison to a control group. To that end, we developed an online questionnaire specifically tailored to examine these hypotheses. Following approval of this procedure by the same University Institutional Review Board (n° 1468/CEIH/2020), participants were contacted by email on June 1, 2020, 1 week after they concluded their final exams, and were invited to complete the aforementioned survey no later than June 15, 2020. As a further incentive, two tablets were raffled among all participants who completed their participation in the entire longitudinal investigation. The online questionnaire included a preliminary note informing participants that any of their potential responses to the questions were optional and not contingent on receiving any of the incentives that they had been offered. From the initial sample of 197 participants, a total of 97 participants, 44 participants from the CRAFT program group and 53 participants from the control group, filled in the online survey.

##### Setting and participants

Participants were higher education student musicians of the Royal Conservatory of Music Victoria Eugenia, a public institution located in Granada, Spain, providing an advanced 4-year higher education music curriculum equivalent to a bachelor’s degree. Inclusion criteria required participants to be full-time student musicians aged 18 years or older enrolled at the Royal Conservatory of Music Victoria Eugenia during the academic year 2019/2020. Students were excluded from the study if they received any psychological, psychiatric, and/or psychopharmacological help during the lockdown. Four participants in the CRAFT program group were excluded from the study following this criterion. Thus, the final total sample for the current study consisted of 93 participants – 40 participants in the CRAFT program group and 53 participants in the control group – age range 18–39 years, of whom 55 were females.

##### CRAFT program protocol

Participants in the CRAFT program group received a total of 23 h of instruction in the program, which corresponded to the total curriculum time allotted for each one of the CRAFT-based elective subjects (Mindfulness and Emotional Intelligence). The first 16 classes were taught in a multi-purpose and spacious classroom well-equipped with yoga mats, blocks, and other materials to facilitate the performance of the different practices implemented. Due to the lockdown, the last 7 h were completed through the online platform *Zoom*. The entire instruction was administered by the creator of the CRAFT program at a frequency of delivery of once a week for 1 h per subject. Beyond her academic formation as a professional musician, translator, and Ph.D. in phonetics applied to singing, the founder of the program is an advanced certified yoga teacher who holds a postgraduate degree in Expert on Mindfulness in the Educational Context. Her mind–body-related background encompasses over 30 years of meditative practice from both ancient Buddhist and yogic traditions, 26 years as a yoga practitioner, and over 15 years of both independently teaching these practices and integrating them within her academic area of professional expertise as a professor of the Royal Conservatory (i.e., music and language higher education). The developer of the program (author MPP) was blind to the study hypotheses, measures, and participants’ recruitment.

Though the program was applied in both elective subjects, there was a preponderance of yoga and mindfulness content in the CRAFT-based elective subject of mindfulness, whereas a prevalence of emotional intelligence, compassion, and positive psychology theory and practice was at the core of the CRAFT-based elective subject of Emotional Intelligence. Another notable difference was that the 15-min physical protocol based on *hatha* yoga was only applied in the CRAFT-based elective subject of mindfulness. Despite these differences, both the five elements of the program and the four foundations were taught in both subjects. During the first 13 classes, there was a focus on working on the first, second, and third elements of the program (e.g., consciousness, relaxation, and attention), whereas for the remaining 10 classes, all elements of the program were taught and practiced.

The five elements of the program are worked through different practices based on its four foundations and adapted to the specific needs of student musicians. Therefore, particular emphasis is given through reflective group discussions and debates on how to specifically integrate them within their daily lives to address their academic, professional, and life demands. For instance, the first element *Consciousness* is first developed through the cultivation of observing one’s body, mind, and emotions with openness, compassion, and a non-judgmental attitude. One of the main goals of *Consciousness* is that student musicians become more aware of their physical, mental, and emotional states so that they can self-regulate them appropriately to meet their particular needs. This is trained through both *hatha* yoga and mindfulness-based practices such as *antar mouna*, breath awareness, RAIN (i.e., recognize, accept, investigate, non-identification), yoga postures, meditation, and other related practices created by the developer of the program such as the FEM meditation (i.e., meditation based on the impartial observation of physical sensations, emotions, and thoughts). Hence, student musicians are taught how to implement such practices not only to increase their awareness but also to integrate them in their daily lives to self-regulate their posture, music performance anxiety, perfectionism, musculoskeletal problems, and other health and well-being concerns. This fusion of both mindfulness and yoga leads to a CRAFT mindful yoga that is also applied in the remaining elements of the program. Thus, the second element *Relaxation* was developed through various *pranayamas* (e.g., *sitali*, *sitkari*, *bhramari*, alternate nostril breathing, and full yogic breathing), visualization techniques, yoga *nidra*, *shamatha* meditation, and other *hatha* yoga-based relaxation practices including progressive-muscle relaxation and autosuggestion components. The third element *Attention* was mainly worked through different meditation techniques (e.g., mantra meditation, open monitoring meditations, breath awareness meditation, and attention-based exercises and games), and specific *pranayama* practices. The path of *raja* yoga, with a focus on its last three meditative components (*dharana*, *dhyana*, and *samadhi*), is discussed and practiced along with other mindfulness-based meditations and practices such as *shamatha* meditation from ancient Buddhist traditions and the body scan from the modern MBSR program. The development of consciousness, relaxation, and attention through these practices paves the way to the fourth element *Happiness*, fostering, in turn, a meditative state of “flow experience”, or the so-called “engagement” component from positive psychology, whereby an optimal music performance might emerge. This is also worked in the classroom through group-meditative improvisations and mindful concerts to tune student musicians into a state of full absorption, self-control, and happiness. In addition to the practices of mindfulness and yoga, which share the profound purpose of bringing everlasting happiness among its practitioners, the teaching of the fourth element *Happiness* added intrinsic components from both positive psychology and emotional intelligence. Some of these were gratefulness (e.g., listing grateful things and events), engagement (e.g., flow experiences through meditative improvisations), and emphasizing one’s strengths and values from positive psychology; or the cultivation of empathy from emotional intelligence. Under the four foundations, happiness was studied from both the Hedonic and the Eudemonic perspectives, giving more emphasis on the latter. Moreover, other specific mindfulness practices such as loving-kindness meditation (e.g., *metta* meditation and *tonglen*) were used to teach self-compassion, compassion, and unconditional love as a pathway to develop happiness. Lastly, in close connection with the first and fourth elements *Consciousness* and *Happiness*, but also relying on the lead-up work to develop *Relaxation* and *Attention*, students received instruction in the fifth element *Transcendence*. Thus, the understanding of transcendence at an experiential level requires the mastery of all previous elements to be able to tune into the ultimate transcendental pursuits of self-knowledge and self-inquiry. It is a journey of finding meaning to life and true happiness rooted in the self rather than on the fleeting external reality. Through this element, students learn the ability to see things from various perspectives, dis-identifying themselves from their immediate ever-changing realities and concerns, and ultimately transcend them through a self-transforming attitude. This fifth element fosters creativity – as a crucial mechanism for transforming and generating new perspectives, possibilities, situations, and realities – as well as the four strengths of positive psychology related to transcendence: the appreciation of excellence and beauty, gratitude, hope, sense of humor, and spirituality (i.e., finding meaning to life). Other important aspects worked through transcendence are the promotion of intention setting, harmonious passion, and *karma* yoga (i.e., selfless service) as key instruments to flourish as individuals and as a society. Practices from all four foundations, with a focus on meditative practices based on both yoga and mindfulness, as well as reflective group discussions on their various philosophical underpinnings and real-life applications were used to teach this element.

A CRAFT program protocol, adapted to and implemented with higher education language students enrolled at the Faculty of Translation and Interpretation of the University of Granada, Spain, has been registered in Clinical Trials (NCT04392869). A further detailed description of the course outline for the CRAFT-based elective subjects of mindfulness and emotional intelligence during the academic year 2019–2020 including objectives, content, practices, and other program procedures per session can be accessed in the Supplementary Material file linked to the current study. In addition, further specifications of the foundations, elements, and practices of the program can be found elsewhere (Posadas, 2019; Posadas and Bartos, under review). All participants in the CRAFT program group were encouraged to complete at least 2 h of home-based practice weekly. This included practicing at least three times a week the following components: the physical protocol, only in the CRAFT-based subject of mindfulness; both formal and informal meditation; and keeping a journal to document their own experiences with the practice of the program. A compulsory attendance rate of 80% was required to complete their instruction in the CRAFT-based elective subjects of mindfulness and emotional intelligence. In the sample for the present survey administered during the lockdown, all participants in the CRAFT program group had met this minimum attendance requirement and can thus be said to have successfully completed the program.

### Measures

#### Online Questionnaire

The online questionnaire included seven questions. The first question asked participants whether they received any psychological, psychiatric, and/or psychopharmacological help. The second question asked participants whether they experienced any severe setback due to the COVID-19 pandemic and lockdown situation. The third question asked participants whether they had experienced relevant changes in their health and well-being due to the exceptional COVID-19 situation, while the fourth question invited participants to indicate those changes in case of reporting an affirmative answer to the third question. The fifth question included three separate statements asking participants how they would rate on a 10-point visual analogue scale their physical, psychological, and sleep quality during the lockdown. The sixth and seventh questions were specifically related to the current study hypotheses. The sixth question asked participants whether they had implemented any particular practice or activity learned throughout the academic year to improve their physical, mental, and emotional state during the lockdown. Lastly, the seventh question invited participants to indicate, in case of reporting an affirmative answer to the sixth question, which particular practice or practices they implemented, where they learned it, and briefly describe their own experience with it and the impact they had on their physical, mental, and emotional state.

### Data Analysis

Data were analyzed using IBM SPSS v. 25.0 (Armonk, NY, United States: IBM Corp. 2017). Descriptive statistics were used to summarize all variables using means, standard deviations, counts, proportions, and frequencies. To determine differences between groups, separate two-way chi-square tests of independence and one-way ANOVAs were conducted for all categorical and continuous variables, respectively.

## Results

To hone the rigor of these results in terms of addressing the current study hypotheses, we conducted analyses for both the total sample and a filtered sample after excluding those participants that reported previous experience with yoga and/or meditation other than in the CRAFT program before the onset of the lockdown and/or in the CRAFT program during the academic years 2017–2019. This was to ensure any potential effects of prior familiarization were considered. The participants’ demographic characteristics for both samples across the experimental and control groups are displayed side by side in Table 1. As can be observed from the total sample, the filtered sample was the result of excluding 31 participants who reported previous yoga and/or meditation experience, 9 participants (22%) from the CRAFT program group, and 22 participants (41%) from the control group. The evidence of a marginally significant association of previous yoga/meditation experience by group, emphasized by a higher observed frequency of controls, supported further the need of controlling for such potential influence by presenting additional analyses conducted on a filtered sample. There were no significant differences for any of the other demographic variables between groups within both samples, except for grade year, whereby the highest proportion of participants in the control group were distributed among the first- and second-grade year, as opposed to the predominance of third-grade year participants in the CRAFT program group.

**TABLE 1:** Participants’ demographic characteristics.

Variables	Total sample			Filtered sample^a^		
	CRAFT program group (*n* = 40)	Control group (*n* = 53)	*p*	CRAFT program group (*n* = 31)	Control group (*n* = 31)	*p*
Age	22.55 ± 3.79	22.38 ± 4.64	0.85	22.71 3.94	22.81 ± 5.12	0.93
Years of musical practice	13.80 ± 3.30	13.26 ± 3.74	0.41	13.98 ± 3.59	12.95 ± 3.57	0.26
Gender						
Females	23 (57%)	32 (60%)	0.78	17 (55%)	17 (55%)	1
Level of education			0.49			0.28
High school	31 (77%)	46 (87%)		24 (77%)	27 (87%)	
Bachelor’s degree	8 (20%)	5 (9%)		6 (19%)	2 (6%)	
Master’s degree	1 (2%)	2 (4%)		1 (3%)	2 (6%)	
Grade year			**0.003**			**0.022**
First	5 (12%)	20 (38%)		4 (13%)	12 (39%)	
Second	9 (22%)	18 (34%)		7 (23%)	10 (32%)	
Third	18 (45%)	9 (17%)		15 (48%)	5 (16%)	
Fourth	8 (20%)	6 (11%)		5 (16%)	4 (13%)	
Yoga/meditation experience	9 (22%)	22 (41%)	0.05	NA	NA	
CRAFT (2017–2019)	1 (2%)	7 (13%)	0.07	NA	NA	
Other yoga/meditation	8 (20%)	15 (28%)	0.36	NA	NA	

*Values are mean ± SD with respective p values computed with one-way ANOVA, or n (%) with respective p values computed with chi-square. Bold font denotes significance. ^a^Filtered sample after excluding participants that reported previous exposure with yoga/meditation other than in the CRAFT program before the lockdown and in the CRAFT program during the academic years 2017–2019. NA = not applicable.*

The results for the health and well-being online questionnaire by groups within both samples are displayed in Table 2. No significant differences between groups were found in terms of severe COVID-19 setbacks, and health and well-being changes, within both samples. A large number of participants in both samples, 72 and 60% in the CRAFT program and control groups, respectively, within the total sample, and 71 and 74% in the filtered sample, reported having experienced relevant changes in their health and well-being. Among these, mental–emotional problems were the most frequently reported. Within the total sample, 52 and 51% of participants in the CRAFT program and control groups respectively suffered from these disturbances, while in the filtered sample, these proportions were slightly higher, 55 and 64%, respectively. In this domain, it appears that stress and anxiety were the most common concerns across both groups. In addition, 35 and 19% of participants in the CRAFT program and control groups, respectively, within the total sample, and 35 and 19% within the filtered sample, indicated affection from physical disturbances. Lastly, for this section of health- and well-being-related variables, separate one-way ANOVAs analyses revealed that there were no significant differences between groups across samples for quality of sleep and physical and mental–emotional health states.

**TABLE 2:** Health and well-being self-reported changes and behaviors across groups during the lockdown.

Perceived outcomes	Total sample			Filtered sample^a^		
	CRAFT program group (*n* = 40)	Control group (*n* = 53)	*p*	CRAFT program group (*n* = 31)	Control group (*n* = 31)	*p*
Severe COVID-19 setbacks	4 (10%)	5 (9%)	0.93	5 (16%)	2 (7%)	0.23
Health and well-being Changes	29 (72%)	32 (61%)	0.22	22 (71%)	23 (74%)	0.78
Positive changes	6 (15%)^b^	3 (6%)^b^	0.13	4 (13%)^b^	3 (10%)^b^	0.69
Mental–emotional issues	21 (52%)^b^	27 (51%)^b^	0.88	17 (55%)^b^	20 (64%)^b^	0.43
Anxiety	13 (32%)^b^	10 (19%)^b^	0.13	10 (32%)^b^	9 (29%)^b^	0.78
Stress	13 (32%)^b^	14 (26%)^b^	0.52	10 (32%)^b^	12 (39%)^b^	0.60
Fear, worry, and despondency	6 (15%)^b^	6 (11%)^b^	0.60	6 (19%)^b^	5 (16%)^b^	0.74
Physical issues	14 (35%)^b^	10 (19%)^b^	0.08	11 (35%)^b^	6 (19%)^b^	0.15
Tiredness	6 (15%)^b^	3 (6%)^b^	0.13	6 (19%)^b^	2 (6%)^b^	0.13
Sleep disturbances	4 (10%)^b^	3 (6%)^b^	0.43	3 (10%)^b^	2 (6%)^b^	0.64
10-point VAS health states (1–10)						
Physical state	6.80 ± 1.83	6.92 ± 1.97	0.76	6.97 ± 1.83	6.90 ± 2.02	0.90
Mental and emotional state	6.23 ± 1.69	5.98 ± 2.2	0.55	6.03 ± 1.68	5.87 ± 2.41	0.76
Sleep quality	5.93 ± 2.4	5.91 ± 2.50	0.97	6.06 ± 2.46	6.00 ± 2.57	0.92
Engagement in health practices	37 (92%)	31 (58%)	** < 0.001**	29 (93%)	17 (55%)	** < 0.001**
Yoga/meditation practices	35 (95%)^b^	6 (19%)^b^	** < 0.001**	27 (93%)^b^	2 (12%)^b^	** < 0.001**
CRAFT	25 (68%)^b^	0 (0%)^b^	NA	21 (72%)^b^	0 (0%)^b^	NA
Other yoga/meditation	13 (35%)^b^	6 (19%)^b^	0.15	7 (24%)^b^	2 (12%)^b^	0.31
Other practices	18 (49%)^b^	29 (93%)^b^	** < 0.001**	15 (52%)^b^	15 (88%)^b^	**0.012**
Alexander technique	2 (5%)^b^	8 (26%)^b^	**0.018**	2 (7%)^b^	4 (23%)^b^	0.11
Exercise	17 (46%)^b^	23 (74%)^b^	**0.018**	14 (48%)^b^	12 (71%)^b^	0.141
Unspecified	0 (0%)^b^	1 (3%)^b^	0.27	0 (0%)^b^	0 (0%)^b^	NA
Perceived benefits from practices	29 (78%)	16 (52%)	**0.020**	23 (79%)	7 (41%)	** < 0.01**
Yoga/meditation	26 (70%)^b^	1 (3%)^b^	** < 0.001**	20 (69%)^b^	0 (0%)^b^	** < 0.001**
CRAFT	19 (51%)^b^	0 (0%)^b^	** < 0.001**	16 (55%)^b^	0 (0%)^b^	** < 0.001**
Other yoga/meditation	10 (27%)^b^	1 (3%)^b^	**0.008**	5 (17%)^b^	0 (0%)^b^	0.07
Other practices	12 (32%)^b^	15 (48%)^b^	0.18	10 (34%)^b^	6 (35%)^b^	0.96
Alexander technique	0 (0%)^b^	5 (16%)^b^	**0.011**	0 (0%)^b^	3 (18%)^b^	**0.02**
Exercise	12 (32%)^b^	9 (29%)^b^	0.76	10 (34%)^b^	3 (18%)^b^	0.22
Unspecified	0 (0%)^b^	1 (3%)^b^	0.27	0 (0%)^b^	0 (0%)^b^	NA

*Values are mean ± SD with respective p-values computed with one-way ANOVA, or n (%) with respective p-values computed with χ^2^. Bold font denotes significance. All displayed percentages refer to the total number of participants within group except for the variables related to the practices implemented and their perceived benefits whereby they refer to the total number of proactive participants within group; ^a^Filtered sample after excluding participants that reported previous exposure with yoga/Meditation other than in the CRAFT program before the lockdown and in the CRAFT program during the academic years 2017-2019. ^b^Percentages from non-mutually exclusive categories. NA = Not applicable.*

Our first hypothesis was that there would be a higher proportion of participants in a CRAFT program group than in a control group that would be proactive in terms of implementing practices as coping strategies to improve their health and well-being during the lockdown. The two-way chi-square analyses revealed that there was a significant association between participants’ group (experimental versus control) and their activity in terms of implementing practices to improve their health and well-being, in both the total sample, χ^2^(1) = 13.41, *p* < 0.001, and the filtered sample, χ^2^(1) = 12.13, *p* < 0.001. The significance level in this association was evident by the high proportion of proactive participants in the CRAFT program group, 92 and 93%, in contrast to the 58 and 55% of their counterpart controls in the total and filtered samples, respectively.

As can be observed from the last section of Table 2, proactive participants across both groups and samples engaged in the following four types of practices – either exclusively to one or concurrently to more than one – to improve their health and well-being during the lockdown: Yoga and/or meditation practices learned in the CRAFT program, other Yoga and/or meditation practices either identified as different from the CRAF program or not linked to it, exercise of different types, and Alexander technique-based practices learned in the elective subject ergonomics. Additional separate chi-squared analyses showed that, except for exercise and Alexander technique-based practices in the filtered sample and other yoga/meditation in both samples, participants’ engagement in each of the different types of practices was not independent of their group participation. Consistently across both samples, there was a higher proportion of participants applying yoga and/or meditation practices (CRAFT and other yoga/meditation) in the CRAFT program group than in the control group, while the proportion of participants employing other practices (exercise and Alexander technique-based practices together) in the control group was higher compared to the CRAFT program group.

As expected, there was a higher proportion of CRAFT-based practice implementation than other types of practices in the CRAFT program group. In the total sample, yoga and/or meditation practices from the CRAFT program were implemented by 68% of participants followed by exercise, 46%, other yoga/meditation, 35%, and Alexander technique-based practices, 5%. In the filtered sample, a similar pattern of results was observed as yoga and/or meditation CRAFT-based practices were implemented by 72% of participants, whereas exercise and Alexander technique-based practices were implemented by 48 and 7% of participants, respectively. Therefore, these findings notably support the applicability of the CRAFT program during the lockdown. Conversely, exercise-based activities were distinctly the most utilized practices among participants in the control group, in both the total and filtered samples, respectively, 74 and 71%, though some participation in other yoga/meditation and Alexander technique-based practices was also evident.

The second hypothesis was that the proportion of proactive participants perceiving benefits from their practices implemented would be higher in a CRAFT program group than in a control group. The two-way chi-square analyses showed that there was a significant association between participants’ acknowledgment of perceived benefits from their practices implemented and their group (experimental versus control), in both the total sample, χ^2^(1) = 5.399, *p* =0.020, and the filtered sample, χ^2^(1) = 6.870, *p* = 0.009. Notably, 78 and 79% of the proactive participants in the CRAFT program group reported perceived benefits as compared to the 52 and 41% of their counterpart controls in the total and filtered samples, respectively.

As evidenced by separate chi-square analyses conducted in both samples, participants’ perceived benefits from their practices implemented were not independent of their group participation, except for other yoga/meditation practices in the filtered sample, and exercise and other practices (exercise and Alexander technique-based practices) in both the total and filtered sample. The most noticeable difference was that the proportion of participants acknowledging perceived benefits from yoga and/or meditation practices was higher in the CRAFT program group than in the control group. By contrast, the proportion of participants perceiving benefits from the Alexander technique-based practices was higher in the control group than in the CRAFT program group, though this analysis was conducted on a very limited number of responses. Interestingly, despite the fact that there was a higher proportion of proactive participants exercising in the control group than in the CRAFT program group, there was no difference in the proportion of participants perceiving benefits from it between both groups within both samples. This result raises questions as to whether participation in the CRAFT program group was influential for those participants in this group who implemented exercise practices, either in conjunction with practices learned in the program or not, in terms of how they were applied, valued, and/or perceived.

As expected, there was a higher proportion of proactive participants in the CRAFT program group perceiving benefits from their engagement in CRAFT-based practices than from other types of practices. In the total sample, 51% of the proactive participants in the CRAFT program group acknowledged perceived benefits from implementing yoga and/or meditation practices learned in the program, followed by 32% from exercise activities, and 27% from other yoga and/or meditation practices. Similarly, in the filtered sample, 55% of proactive participants perceived benefits from implementing CRAFT-based practices, 34% from exercising, and only 17% from other yoga and/or meditation practices. By contrast, among proactive participants in the control group, 29 and 16% of participants in the total sample reported perceived benefits from the application of exercise and Alexander technique-based practices, respectively, whereas an equal distribution relative to such proportions was observed in the filtered sample (18% from exercising and 18% from the application of Alexander technique-based practices).

**FIGURE 1:**
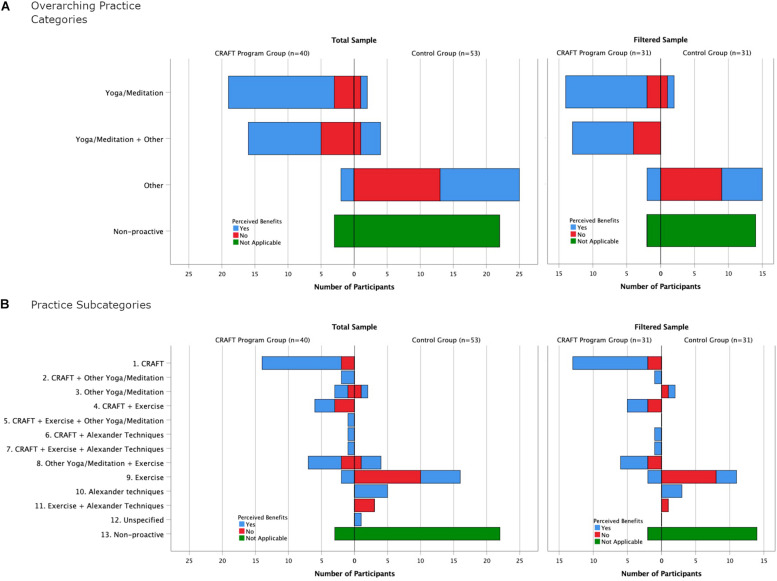
The relationship between participants’ engagement in the various overarching and subcategories of practices during the lockdown and their perceived benefits across groups and samples [**(A)**: overarching practice categories; **(B)** practice subcategories]. In the filtered sample, those participants that reported previous experience with yoga and/or meditation other than in the CRAFT program before the onset of the lockdown and/or in the CRAFT program during the academic years 2017–2019 were excluded. Subcategories 1, 2, and 3 belong to the overarching category “Yoga/Meditation”; Subcategories 4, 5, 6, 7, and 8 belong to the overarching category “Yoga/Meditation + Other”; Subcategories 9, 10, 11, and 12 belong to the overarching category “Other.”

In the manner in which the aforementioned results are presented, i.e., through non-mutually exclusive representation, the proportion of participants who relied on implementing just one type of practice alone cannot be discerned, or how many participants opted for a particular combination of practices. To satisfy the provision of such perspective, Figure 1 illustrates the results of this study in a mutually exclusive representation, involving overarching practice categories (Figure 1A) and subcategories yielded from the various combinations of the four types of practices implemented (Figure 1B) along with their perceived benefits. Again, a clear pattern emerged, illustrating that participants in the CRAFT program were more likely to engage in yoga/meditation practices and more likely to report perceived benefits from those practices. When excluding participants who had reported prior experience with yoga and meditation (filtered sample on right-hand side), a very similar pattern was evident.

Table 3 includes some of the quotes illustrating some of the perceived benefits underlined by participants within their respective groups from the various categories of practices they had implemented. Answers by those students in the CRAFT program who had practiced yoga/meditation referred to the fact that they had learned skills in the CRAFT program, while some participants in the control group were drawn to audiovisual material sourced from the Internet. Some other practices by the control group overlapped to some degree with meditation, although they appeared to have been conducted without the systematic structure that formalized mindfulness and meditation practices typically have.

**TABLE 3:** Participants’ perceived benefits from practices implemented to improve their health and well-being during the lockdown across groups and samples.

Practices implemented	Exemplar responses from proactive participants					
	Craft program group (*N*_*TS*_ = 37; *N*_*FS*_ = 29)	Beneficial effect		Control group (*N*_*TS*_ = 31; *N*_*FS*_ = 17)	Beneficial effect	
		*N*_*TS*_ (%)	*N*_*FS*_ (%)		*N*_*TS*_ (%)	*N*_*FS*_ (%)
Yoga/Meditation	“I have practiced Meditation. I have learnt it in the Emotional intelligence class and it has been an important element to bear the situation. The changes have been relevant. Management of anxiety, stress, interfamily relationships, time management. It has been an essential help in multiple aspects.”	16 (43)	12 (41)	“Yoga learnt through video lessons on YouTube. Honestly, I have noticed certain improvement from the first practice though I have been doing it for not a long time to feel more important beneficial effects.”	1 (3.2)	1 (6)
	“The various techniques learnt in the Mindfulness subject such as the physical Protocol, Meditations, breath control helped me live with the current situation and see it with a neutral perspective.”					
Yoga/Meditation + Other	“Sports made me liberate accumulated tensions and feel much better with myself. I meditated a lot and thanks to the subjects of Mindfulness and Emotional intelligence I am able to manage and self-regulate my emotions, I am more conscious of my thoughts, and my sate in general.”	11 (30)	9 (31)	“Guided meditations that helped me relax during anxious moments. Sports helped me feel my body stronger.”	3 (9.7)	0 (0)
				“Meditation (audio from authorized courses), cardio, push-ups, abs, squats. All of this has helped me bear the effects I have described but I also believe that in a lockdown state like this there is no one who can be healthy regardless of what they do.”		
	“Every day I did physical activity that helps me to relax/calm down physically, mentally, and emotionally. I also practiced meditation but not as much as I would have liked. It was difficult as it requires a prior state of concentration and calmness. However, when I applied it I have noticed outstanding changes.”					
Other	“Online training with a personal training through Instagram. It has been very positive because it was helping me to unleash my energy and to put my body in operation”.	2 (5)	2 (7)	“Only doing sports has helped me keep active, feel more energic and be more positive about looking at things.”	12 (38.7)	6 (40)
				“I used the ‘conscious rest’ practice from the ergonomics subject when I was overwhelmed and could not take it anymore. Resting for 5–10 min and let myself go without thinking of anything gave me tranquility and relaxation, which I needed it.”		
	“Sports five times a week and I am very proud. I have not experienced a lot of change but it makes me feel well”.					

*N_TS_ = Number of proactive participants in the total sample; N_FS_ = Number of proactive participants in the filtered sample after excluding participants that reported previous exposure with yoga/Meditation other than in the CRAFT program before the lockdown and/or in the CRAFT program during the academic years 2017–2019.*

## Discussion

The current study explored the applicability and perceived impact of the CRAFT program to improve higher education student musicians’ physical, mental, and emotional health and well-being during the lockdown in comparison to a control group. To increase the accuracy and breadth of this exploration, data analyses were conducted on both the total sample and a filtered sample excluding those participants who had reported previous experience with any yoga and/or meditation training other than in the CRAFT program before the onset of the lockdown, and/or in the CRAFT program during the academic years 2017–2019.

Our results showed that student musicians’ participation in a CRAFT program group was associated with higher engagement in terms of implementing practices to improve their health and well-being than in a control group. This significant association was met for both the total and filtered sample and indicated that participants following the CRAFT program training showed a more proactive attitude than participants in the control group in terms of applying strategies to improve their health and well-being. The most conspicuous observation to emerge from this analysis was that almost half of the participants in the control group were non-proactive, whereas all but just three participants were proactive during the lockdown in the CRAFT program group. A meaningful comprehension of these results requires further consideration of the type of practices implemented and whether these align with the CRAFT program procedures and foundations. Remarkably, the vast majority of the proactive participants in the CRAFT program group, 95%, utilized yoga and/or meditation practices as opposed to the 19% of their counterpart controls. Furthermore, we found from the filtered sample that for up to 20 participants of the CRAFT program group, their yoga and/or meditation practices were those learned in the CRAFT program, while 13 participants relied exclusively on this exposure to improve their health and well-being. Therefore, these figures clearly show not only the preponderance of yoga and/or meditation practices as coping strategies in the CRAFT program group in comparison to the control group, but also the influence that the exposure to the CRAFT program made in terms of making such a difference. One of the key foundations of the CRAFT program is yoga. As underlined by Desikachar et al. (2005), an important characteristic of yoga is that it promotes a self-caring attitude by encouraging the practitioners to be actively involved and responsible of their own health. In addition, the authors explained that yoga’s approach to the health–ill conundrum is unique by attempting to find the root of the problem within and potentially addressing each individual’s needs through specific practices. These two principles engrained in the yoga lifestyle, which are also fostered as part of the CRAFT instruction, might have raised student musicians’ consciousness of their inner responsibility – to a greater extent than those not attending the program – for taking actions towards improving their health and well-being during the lockdown.

In addition, among proactive participants in both the total and filtered samples, participation in a CRAFT program group was associated with higher rates of perceived benefits from the practices implemented in comparison to a control group. This result was highlighted not only by the acknowledgment of a beneficial effect from most of the participants in both the total (19 out of 25) and filtered sample (16 out of 21) implementing yoga and/or meditation practices learned in the CRAFT program, but also by the absence of significant differences between groups in the proportion of participants who indicated perceived benefits from exercising. Remarkably, in the CRAFT program group, most participants exercising in both the total (12 out of 17) and filtered (10 out of 14) samples stated perceived benefits from it, while such indication was only acknowledged by some participants in the control group in the total sample (9 out of 23) and just a few in the filtered sample (3 out of 12). Thus, it could be arguably postulated that participation in a CRAFT program might not only have been valued and applied meaningfully to real-life situations, but it could also have had an effect on how these practitioners engaged in, lived, perceived, and appreciated their experience with other practices they implemented, which might also have led to a series of unexpected benefits. The aforementioned rationale finds intrinsic alignment with the four foundations of the CRAFT program and their respective investigated and hypothesized benefits. For instance, components of positive psychology such as gratitude and savoring encourage people to appreciate and value the activities they engage in, which subsequently has been linked to further benefits such as increased happiness, well-being, resilience, positive affect, and psychological well-being (Sin and Lyubomirsky, 2009; Martínez-Martí et al., 2010; Mongrain and Anselmo-Matthews, 2012; Smith and Hanni, 2019). In addition, the concept of savoring mirrors to a great extent the concept of engagement from the PERMA model of positive psychology (Seligman, 2011) and Csikszentmihalyi’s (1990) construct of flow, which are related to the development of a heightened state of awareness, and higher self-control and meaningfulness of one’s experiences (Csikszentmihalyi, 1990). Similarly, both yoga and mindfulness-based practices, which are highly grounded on cultivating awareness of one’s inner physical, mental, and emotional states and developing a mindful and attentive attitude in one’s actions and behaviors (Gard et al., 2014; Tang et al., 2015; Guendelman et al., 2017), have been associated to the promotion of flow states, gratitude, and meaningful living (Ivtzan and Papantoniou, 2014). Therefore, such abilities, if practiced and developed under proper and experienced guidance, could automatically be transferred to other activities, potentially enhancing not only their original effects but also the way these could be perceived and valued.

Moreover, the proportion of participants perceiving benefits from CRAFT-based practice implementation was higher than the proportion of participants perceiving benefits from other types of practices. Likewise, the proportion of participants engaging in CRAFT-based practices was higher than the proportion of participants engaging in other types of practices to improve their health and well-being during the lockdown. These results, taken together with those reported for the main hypotheses of the current study, were further replicated in the filtered sample after controlling for previous yoga and/or meditation experience. Thus, our findings suggest that participants acknowledged the usefulness and effectiveness of the yoga and/or meditation components of the CRAFT program out of their formal academic instruction and, potentially, to a greater extent than other types of practices they implemented, during stressful circumstances elicited by the pandemic. Some of the main benefits outlined by participants involved in the CRAFT-based elective subjects of mindfulness and emotional intelligence were that the practices helped them relax, clear their minds, relieve from stress and anxiety, cope with the lockdown situation, feel better, self-regulate their emotions, deal with their family relationships, become more conscious, achieve greater concentration, develop a positive attitude, manage their time, and see things from another perspective. These perceived benefits are in close connection with the five elements of the CRAFT program (i.e., consciousness, relaxation, attention, happiness, and transcendence) and concur well with the investigated benefits of the four foundations they emerge from (i.e., yoga, mindfulness, positive psychology, and emotional intelligence). However, proactive participants in the control group also cited positive effects from their engagement in exercise or the Alexander technique-based practices learned in the elective subject “ergonomics”, which suggested that these activities might have also played an important role in helping them cope with the lockdown demands and related health and well-being concerns.

In this study, the impact of the COVID-19 lockdown on the health and well-being of music conservatory students was associated with a prevalence of mental–emotional concerns such as stress and anxiety. This was in conformity with recent studies reporting a high incidence of such disturbances among confined higher education students (Elmer et al., 2020; Huckins et al., 2020; Kaparounaki et al., 2020; Li et al., 2020; Liu et al., 2020; Odriozola-González et al., 2020). Moreover, physical problems were also prevalent in our sample. However, the occurrence of health and well-being concerns did not differ among groups, or how participants rated their physical, mental–emotional, and quality of sleep states. Hence, these results were unexpected and raised concerns as to whether the higher engagement observed from the implementation of the CRAFT program to improve these states was efficacious in comparison to a control group. Notwithstanding, although the cross-sectional design of this lockdown-specific study did not allow us to determine whether any between-group differences happened due to the program, it should be acknowledged that the low dosage of program delivery of 50 min per week and the emerging difficulties from adapting it to a remote learning education might have compromised its actual effect and application. In addition, as mentioned above, the control group also included proactive participants who reported perceived benefits from practicing exercise and Alexander technique-based activities to improve their health and well-being and their potential influence cannot be underestimated.

### Limitations, Strengths, and Further Research

Despite partially meeting our initial hypotheses, these findings should be taken with caution due to the existence of several limitations, some of which have been already outlined at the end of the preceding paragraph. First and foremost, the results of the current study are interpreted from the analyses of self-reported responses collected at just one point in time. Therefore, they remained at a perceptual level and no causation could be established from their cross-sectional examination. This, in turn, outlines a further limitation for the absence of any objective measure to substantiate these findings. Another limitation was that participants’ allocation to groups was based on their own enrolment choices and preferences. This is often stressed as a source of bias as it contemplates the possibility that participants are already familiar with or naturally gifted for the abilities imparted in the programs they enrolled in. Although we acknowledged such possibility and attempted to partially control for it by including a filtered sample, we also underlined that self-selection is what normally happens in the community, having thus a value on its own for it represents reality as it is. Therefore, further studies should also consider this type of naturalistic research, along with traditional randomized control trials, to examine the effectiveness of wellness programs for the health and well-being of our communities, and specifically with higher education students and student musicians. Furthermore, given the holistic approach of the CRAFT program, the use of expanded qualitative methods such as semi-structured interviews could have added further insights into the lived experience and perceived benefits of participants following a health and well-being program during the lockdown. Along these lines, one of the unique aspects underlying the creation of the CRAFT program, from both a programmatic and a research perspective, lies on the basis that combining a careful selection, adaptation, and/or creation of practices and components derived from or inspired by its four foundations – yoga, mindfulness, positive psychology, and emotional intelligence – within a single program could be more complete and effective than a program based on one of them alone. To the best of our knowledge, CRAFT is the first program to synthesize the theories, practices, and philosophical underpinnings of these four disciplines. In this study, we showed how higher education student musicians’ experience with the program was linked to a meaningful real-life application to improve their health and well-being during highly demanding and stressful circumstances (lockdown), even to a greater extent than those not attending the program, in terms of their proactivity and their perceived benefits associated with it. Though these results are promising and provide converging evidence for the social validity of the program, further research is needed to examine the effects of the CRAFT program in comparison with other programs, based on just one of its foundations, within longitudinal pre-post mixed methods designs on various health and well-being domains. Although there were various limitations, this study was strengthened by the addition of a filtered sample that allowed us to control for the potential influence of previous yoga and/or meditation experience; blinding the instructor/developer of the program to the study hypotheses, measures, and participants’ recruitment; the inclusion of incentives; and a detailed description of the program components, practices, and procedures, the specification of which has been repeatedly claimed as a need to be addressed in greater detail when conducting and reporting yoga-based research studies (Sherman, 2012; Elwy et al., 2014; Groessl et al., 2015). We also remind the reader that the purpose of this quasi-experimental study was not to test the efficacy of the CRAFT program but to utilize the opportunity brought about by the COVID-19 pandemic to explore to what extent participants independently perceive the skills taught by the program as beneficial to their health and thus apply them to improve their well-being during the lockdown, and this goal was achieved.

### Relevance to Higher Education and Student Musicians

The results of the current study have important implications, at both educational and health prevention levels, for higher education students of any discipline and specifically for those involved in music studies. Since the COVID-19 pandemic is nowhere near over, and there may be similar pandemics in the future, we urge higher education providers to make the necessary efforts for the promotion of health and well-being programs of this kind, to be implemented within students’ curriculum, to help them cope with the ensuing health and well-being demands. In light of our findings, we encourage that these programs have an emphasis on raising higher education students’ consciousness of the importance of being actively involved in their own health and well-being processes. Particularly, the CRAFT program, as a multifaceted health and well-being program – integrating the theory, practice, and investigated effects of ancient mind–body therapies and modern psychological approaches – offers a variety of practices that seem to be meaningfully applied and transferred to real-life situations with potential benefits. Although much research is still needed, such techniques seem promising to be used by higher education students of any discipline for empowering themselves towards their own life and health and well-being processes, and, as a consequence, as coping or even therapeutic strategies for alleviating various health-related concerns and emotional letdowns; for replacing maladaptive unhealthy behaviors; for preventative care, with the aim of reducing the odds of developing further or more complicated illnesses at later stages of their lives; and for an overall betterment of their daily life and academic needs and performances. Moreover, the high heterogeneity and versatility of the CRAFT program practices and components, targeting various dimensions of health and well-being (e.g., physical, emotional, cognitive, psychological, and spiritual), make it largely accessible to accommodate and embrace diversity among individuals, and hence, potentially suiting the personal needs and/or preferences of higher education students. Specifically, we also endorse the continued application and investigation of the effectiveness of the CRAFT program with higher education student musicians, a population often affected by high levels of perfectionism, musculoskeletal injuries, and music-performance anxiety, and for whom, a harmonious interplay between body, mind, and emotions – through the work of the four foundations and five elements of the program – could be conducive to both maximizing their music performances and improving such aforementioned music-specific concerns (Posadas, 2019).

## Conclusion

The present study showed how higher education student musicians applied at their own will yoga and/or meditation-based learned skills from the CRAFT program as coping strategies in response to the lockdown demands elicited by the COVID-19 pandemic. Despite not being able to determine health and well-being differences attributable to exposure to the program, our findings indicate that curricular participation in a CRAFT program at a music conservatory was associated with greater health and well-being engagement and perceived benefits than controls during the COVID-19 lockdown. This evidence made an even more compelling case for taking preventive measures and implementing more wellness programs including yoga and/or mindfulness components to help higher education students cope with their physical, mental, and emotional health and well-being needs. Therefore, we endorse the continued application of the CRAFT program with higher dosages and frequency of delivery within longitudinal studies conducted on higher education students in the midst of a global pandemic of unprecedented impact. Due to the holistic nature and heterogeneity of the program, further research investigating its influence on the health and well-being of higher education students should incorporate qualitative methods and objective measures within the framework of a robust pre-post mixed methods design with various arms.

## Data Availability Statement

The datasets presented in this article are not readily available because: It was informed in the participants’ consent form that all data collected for this study would be used anonymously for their analysis and further publication in a collective manner, but never individually. The participants recruited in this study represent a small sample from just one institution (Royal Conservatory of Music Victoria Eugenia, Granada, Spain) and from specific reduced courses. Therefore, even considering that participants used an alphanumeric code to safeguard their anonymity, in some instances, they could be potentially identifiable. Nevertheless, any queries about the availability of the datasets to be used for research purposes can be directed to: MF, mjfunes@ugr.es or LB, javier.bartos@autuni.ac.nz.

## Ethics Statement

The studies involving human participants were reviewed and approved by the Institutional Review Board of the University of Granada. The participants provided their written informed consent to participate in this study.

## Author Contributions

LB and CK conceived the quasi-experimental study. MF, MP, and MO secured funding for the larger longitudinal study. MP conducted the program. LB, MF, and MO collected the data. LB, CK, and MF conducted most of the statistical analyses. LB wrote the main draft. All authors contributed to the writing.

## Conflict of Interest

We acknowledge that one of the authors (MP) developed the CRAFT program. However, as stated in the author contributions, MP did not conceive this particular study and also was not responsible for both the data collection and analysis stages of this investigation, which was also a way to manage this particular potential conflict of interest. The remaining authors declare that the research was conducted in the absence of any commercial or financial relationships that could be construed as a potential conflict of interest.
