# Supplementary material for: Developing Resilience During the COVID-19 Pandemic: Yoga and Mindfulness for the Well-Being of Student Musicians in Spain
Source: Front Psychol. 2021 Apr 21;12:642992. doi: 10.3389/fpsyg.2021.642992 (PMC8097029; doi:10.3389/fpsyg.2021.642992)
Supplement: Supplementary file 1 [file Data_Sheet_1.docx]

**BRIEF COURSE OUTLINE**

**2019-2020**

|  | **MINDFULNESS**  **(CRAFT PROGRAMME)** | **EMOTIONAL INTELLIGENCE**  **(CRAFT PROGRAMME)** |
| --- | --- | --- |
| **Introductory**  **module**  Session 1  (hour) | **INTRODUCTORY SESSION**  Introductory session to acquaint the students with the practice of the CRAFT program and its application to higher Music Studies at the Conservatory.  The CRAFT program is a systematic combination of practices derived from ancient philosophies, such as yoga and Buddhism, in conjunction with more recent disciplines such as mindfulness, emotional intelligence, and positive psychology for cultivating and deepening the core human qualities of consciousness, relaxation, attention, happiness, and transcendence.  Introduce the notion of mindfulness, the importance of being present  and its connection with the five elements of the CRAFT program.  Let the students be aware and reflect on the importance of self-engagement and self-responsibility for the development of inner conscious and holistic wellbeing.  Ask the students to introduce themselves to the group through an experiential self-introduction. | **INTRODUCTORY SESSION**  Introductory session to acquaint the students with the practice of the CRAFT program and its application to higher Music Studies at the Conservatory.  The CRAFT program is a systematic combination of practices derived from ancient philosophies, such as yoga and Buddhism, in conjunction with more recent disciplines such as mindfulness, emotional intelligence, and positive psychology for cultivating and deepening the core human qualities of consciousness, relaxation, attention, happiness, and transcendence.  Introduce the notion of emotional intelligence and its 5 components. Learn the relationship between them and the five elements of the CRAFT program.  Let the students be aware and reflect on the importance of self-engagement and self-responsibility for the development of inner conscious and holistic wellbeing.  Ask the students to introduce themselves to the group through an experiential self-introduction. |
| **Module 1**  Session 2 & 3  (2hours) | **CONSCIOUSNESS**  Teach students to be aware of the person as a holistic being. Introduce the notions of consciousness, self-awareness, other-awareness, and inner and outer awareness.  Introduce and explain the concept of «witness consciousness» to observe the threefold relationship of body, mind, and emotion. Delve with the students into the concept of perception.  Explain and examine the body-mind relationship.  Introduce students to FEM meditation, observing impartially body sensations, emotions, and thoughts. Learn specific vocabulary to label the physical, emotional, and mental states.  Introduce students to the first posture of the CRAFT psychophysical protocol with the standing posture of *tadasana*, letting them be aware of their physical and mental postures.  Self-inquiry and reflection about contents and practices | **SELF-AWARENESS & CONSCIOUSNESS**  Teach students to be aware of the person as a holistic being. Introduce the notions of consciousness, self-awareness, other-awareness, and inner and outer awareness.  Introduce and explain the concept of «witness consciousness» to observe the threefold relationship of body, mind, and emotion. Delve with the students into the concept of perception.  Explain and examine the body-mind relationship.  Delve with the students into the 5 components of emotional intelligence, their importance, and their connection with with five CRAFT elements.  Introduce students to FEM meditation, observing impartially body sensations, emotions, and thoughts. Learn specific vocabulary to label the physical, emotional, and mental states  Self-inquiry and reflection about contents and practices |
| **Module 2**  Session 4, 5 & 6  (3 hours) | **RELAXATION-BREATHING-REGULATION**  Let the students examine their thoughts and emotions, and the effects these have on their mind and body.  Introduce the notion of emotions and feelings observing the differences. Let the students examine and explore their basic emotions and the feelings that may arise.  Learn about physical, emotional, and mental relaxation vs. physical, emotional, and mental tension. Delve into the physical and phycological causes and effects of stress.  Introduce breathing as a coping strategy that broadens their inner resources and enhances relaxation and regulation.  Let the students examine the possibility of *responding* instead of *reacting* in different situations.  Ask students to keep a record of their body-mind-emotion perceptions before accomplishing any task during the course  Guide students through *pranayama* and its advantageous effects on the neurocognitive, psychophysiological, respiratory, biochemical, and metabolic functions.  Explore the body-mind standing posture of *tadasana* and respective variants.  Delve into the experience of yoga *nidra* and *samatha* practices.  Self-inquiry and reflection about contents and practices. | **SELF-AWARENESS: CONSCIOUSNESS, PERCEPTION, AND EMOTIONS**  Let the students examine their thoughts and emotions, and the effects these have on their mind and body.  Introduce the notion of basic emotions, the different types and classifications of emotions and feelings, and the basic abilities to process and regulate the emotions.  Introduce the students to differentiate between emotions, feelings, and thoughts and how they can be felt in the body.  Explain and examine the body-mind connection in relation to different emotions and feelings and delve into the ability to observe with equanimity, letting go.  Introduce breathing as a coping strategy that broadens their inner resources and enhances relaxation and regulation.  Let the student examine their strengths and virtues.  Ask students to keep a record of their body-mind-emotion perceptions before accomplishing any task during the course.  Introduce the notion of limiting beliefs and let the student be aware and examine their own limiting beliefs.    Ask students to keep a record of their pleasant and unpleasant experiences.  Guide the students through *shamatha* meditation and internal open-monitoring meditation.  Self-inquiry and reflection about contents and practices. |
| **Module 3**  Sessions 7, 8 & 9  (3 hours) | **MINDFULNESS/FULL ATTENTION**  Introduce the notion of attention deployment and how it can regulate emotional experience.  Let students be aware of the ability and importance of focusing the attention on the present moment and shifting from doing to being.  Train students on attentional regulation and the attitudes of mindfulness. Teach them how they can break unproductive styles of cognitive and emotional processing, and foster positive, proactive ones through formal and informal meditation. The importance of a non-reactive observation. Delve with the students into the ability to *respond* instead of *reacting.*  Introduce students to the Body Scan, mobilizing attention sequentially from body part to body part teaching students to disengage from one part and engage with the new one.  Introduce students to the CRAFT psychophysical protocol (I), letting them be aware of their physical and mental postures.  Guide them through two observation practices: the conscious minute and conscious pleasures.  Self-inquiry and reflection about contents and practices | **SELF-REGULATION, RELAXATION & BREATHING**  Introduce the notion of Full Emotional Intelligence.  Let students be aware of the ability and importance of locating, perceiving, and labeling their emotions.  Train students on emotional abilities and mechanisms to perceive, recognize, understand, and regulate their basic emotions. Teach them how they can break unproductive styles of cognitive and emotional processing, and foster positive, proactive ones through formal and informal meditation. The importance of a non-reactive observation.  Let the students examine the possibility of *responding* instead of *reacting* in different situations.  Let the students examine their limiting beliefs, assumptions, thoughts, emotions, and feelings and the connection with their behavior.  Make them recognize and foster their strengths and virtues to cope with the difficulties and situations they experience.  Ask students to keep a record of their pleasant and unpleasant experiences and observe if they react or respond to them.  Delve with the students into different types of *shamatha* meditation and internal and external open-monitoring meditation.  Self-inquiry and reflection about contents and practices |
| **Module 3**  Sessions 10, 11, 12 & 13  (4hours) | **MINDFULNESS/FULL ATTENTION**  Keep training students on attentional regulation and the attitudes of mindfulness.  Delve with them into the importance of shifting from doing to being and putting it into practice.  Let them examine their scenic presence and their ability to maintain an aligned and mindful posture and a focused mind throughout their musical performances.  Continue practicing the Body Scan meditation, CRAFT psychophysical protocol (I), and the use of as ways to focus attention and increase lung capacity.  Guide students through other types of meditation such as sound meditation (*nada* yoga), Kinhin (walking meditation), Contemplation, or open-monitoring meditation and continue with the complete physical, mental, and emotional conscious relaxation practice of yoga *nidra*.  Self-inquiry and reflection about contents and practices. | **SELF-REGULATION, RELAXATION & BREATHING**  Delve into the notion of Full Emotional Intelligence.  Keep training the abilities of locating, perceiving, labelling, and symbolizing their emotions exploring also their meaning and purpose.  Teach the students the notion of compassion and the importance of a compassionate attitude.  Delve with the students into examining their limiting beliefs, assumptions, thoughts, emotions, and feelings and the connection with their behavior, applying their strengths and virtues to cope with the difficulties and situations they experience in their daily life.  Delve into the causes and effects of stage fright and how to apply all the previous contents to deal with it.  Introduce visualization as a key tool of self-regulation and relaxation technique to deal also with stage fright.  Guide the students into different types of relaxation methods: visualization, creative relaxation, Jacobson´s relaxation, and music therapy.  Self-inquiry and reflection about contents and practices |
| **Module 4**  Sessions 14 & 15  (2hours) | **HAPPINESS/BLISS**  Introduce the notion of flow, happiness, and bliss closely associated with mindfulness, full-awareness, psychological well-being, and meaningful/fulfilling life.  Let the students develop a state of flow during their musical performances.  Reconsider the notion of empathy and forgiveness, and teach students to direct loving-kindness and compassion towards themselves.  Delve with the student into how to cope with perfectionism through the practice of compassion.  Explore new ways of perceiving mistakes and promoting solutions.  Learn about flow as motivation and creativity, and flow as happiness and bliss.  Guide students through observation and *shamatha* & *metta* meditation practices towards themselves.  Continue practicing CRAFT psychophysical protocol (I) and meditation. Introduce the practice of CRAFT psychoemotional protocol as the other side of the same coin to deal with difficult situations.  Start practicing Conscious Listening, Speaking and Watching and Conscious communication applied to working in groups.  Self-inquiry and reflection about contents and practices | **MOTIVATION**  Introduce the concept of positive psychology, its main contributions, findings, and goals.  Introduce the notion of flow, happiness, and bliss closely associated with psychological well-being and a meaningful/fulfilling life.  Examine flow experiences and elements to be present in the state of flow.  Introduce the students to the notion of transcendence, a state that promotes meaning-making to overcome life´s challenges.  Delve into the notion of creativity. Explain the concepts of humor and beauty as by-products of transcendence. Links with motivation and harmonious passion.  Learn about the power of sound and mantra meditation.  Guide the students to examine their motivation tools and abilities to motivate themselves and others.  Explore creative responses to a difficult situation.  Guide them to metta meditation towards themselves and delve into the practice of open-monitoring meditations.  Examine a practical case to observe and analyze the 5 components of emotional intelligence and possible solutions.  Self-inquiry and reflection about contents and practices. |
| **Module 5**  Session 16  (1hour)  **Online Sessions**  17 & 18  (2hours) | **TRANSCENDENCE**  Introduce the students to the notion of transcendence, a state that promotes meaning-making to overcome life´s challenges.  Delve into the notion of creativity. Explain the concepts of humor and beauty as by-products of transcendence. Link these concepts with motivation and harmonious passion. Explain other-centered emotions such as kindness and gratitude; see how they relate to connectedness and communication.  Continue practicing CRAFT psychophysical (I) and psychoemotional protocols and other formal meditations such as *shamatha*-*metta* meditation practice,  meditation for uncertainty and  meditation for stage-fright  Self-inquiry and reflection about contents and practices | **EMPATHY**  Reconsider the notion of empathy and forgiveness, and teach students to direct loving-kindness and compassion towards themselves, towards somebody very close and all beings without distinction.  Delve into the notion of creativity. Explain the concepts of humor and beauty as by-products of transcendence. Links with motivation and harmonious passion. Explain other-centered emotions such as kindness and gratitude; see how they relate to connectedness and communication.  Continue practicing CRAFT psychoemotional protocol and other formal meditations such as *shamatha*-*metta* meditation practice,  meditation for uncertainty and  meditation for stage-fright  Examine practical cases to observe and analyze the 5 components of emotional intelligence and possible solutions.  Self-inquiry and reflection about contents and practices |
| **Module 6**  **Online Sessions**  19 & 20  (2hours) | **COMMUNICATION AS CONNECTION. FROM THE CONSCIOUS SELF**  Introduce the students to communication seen as connection in the context of higher music education.  Encourage the students to embody the “alert-yet-relaxed” mode. Embracing the nerves in the academic context and daily life. Reconsider resilience as a way to be comfortable with being uncomfortable. The key role of motivation and passion.  Practice visualizing before a concert or oral presentation, an exam, or assignment; practice reflecting after, too. Encourage students to be creative and flexible, trying out different approaches, different points of view, and different outcomes. While doing so, help students to practice empathy but feel grounded at the same time.  Continue practicing CRAFT physical (I) protocol and other formal meditations such as *shamatha*, and introduce *metta* and compassion meditation practice (directed to a conflicting person, any person, and all beings without distinction).  Continue guiding them through the observation of flow and creativity activities. Guide them through the observation of mindful pleasures, beauty, humor, gratitude, and the ability to be rooted in the uncertainty.  Self-inquiry and reflection about contents and practices. | **SOCIAL SKILLS:**  **COMMUNICATION AND PERSONAL RELATIONS**  Help students to apply previously learned CRAFT skills in the area of communication and personal relations as in the practice of group performances. Frame communications and the practice of playing together within the broader context of personal relations based on empathy and compassion.  Encourage the students to embody the “alert-yet-relaxed” mode. Embracing the nerves in the academic context and daily life. Reconsider resilience as a way to be comfortable with being uncomfortable. The key role of motivation and passion.  Practice visualizing before a concert or oral presentation, an exam, or assignment; practice reflecting after, too. Encourage students to be creative and flexible, trying out different approaches, different points of view, and different outcomes. While doing so, help students to practice empathy but feel grounded at the same time.  Continue practicing CRAFT psycho-emotional protocol and other formal meditations such as *shamatha*-*metta* meditation practice (directed to themselves, a conflicting person, any person, and all beings without distinction).  Continue guiding them through the observation of flow and creativity activities. Guide them through the observation of pleasant and unpleasant situations with equanimity, appreciation of beauty, humor, gratitude, and the ability to be rooted in the uncertainty.  Examine practical cases to observe and analyze the 5 components of emotional intelligence and possible solutions.  Self-inquiry and reflection about contents and practices |
| **Module 7**  **Online Session**  21  (2hours) | **TIME MANAGEMENT**  Help the students to apply the learned tools and skills for efficient time management.  Delve with the student into a list of tips to plan and organize the time in order to be effective.  Guide them to elaborate their own list of tips for time management.  Self-inquiry and reflection about contents and practices | **TIME MANAGEMENT**  Help the students to apply the learned tools and skills for efficient time management.  Delve with the student into a list of tips to plan and organize the time in order to be effective.  Guide them to elaborate their own list of tips for time management.  Self-inquiry and reflection about contents and practices. |
| **Module 8**  **Online Session**  22  (1hours) | **CRAFT AND THE ART OF MINDFUL, CONSCIOUS AND BLISSFUL LIVING OUR DAILY LIVES**  Experiential training in the CRAFT program for higher musical studies continues and we review and discuss about the pros and cons of the pandemic and how the program could help.  Let them reflect on the notion of obstacles and difficulties as challenges within the academic or professional context and daily life not necessarily as a threat against one's integrity, but as a way to enhance identity, learn and flourish. Our self as a self in process. Link difficulties, obstacles, and life´s challenges to other notions already touched upon in the program, such as creativity, motivation, happiness, and bliss.  Encourage students to make their own selection of the most efficient practices to be applied in their daily life under these special circumstances of COVID-19.  Let students practice on their own. | **CRAFT AND THE ART OF MINDFUL, CONSCIOUS AND BLISSFUL LIVING OUR DAILY LIVES**  Experiential training in the CRAFT program for higher musical studies continues and we review and discuss about the pros and cons of the pandemic and how the program could help.  Let them reflect on the notion of obstacles and difficulties as challenges within the academic or professional context and daily life not necessarily as a threat against one's integrity, but as a way to enhance identity, learn and flourish. Our self as a self in process. Link difficulties, obstacles, and life´s challenges to other notions already touched upon in the program, such as creativity, motivation, happiness, and bliss.  Encourage students to make their own selection of the most efficient practices to be applied in their daily life under these special circumstances of COVID-19.  Let students practice on their own. |
| **Module 9**  **Online Session**  23  (1hour) | **CRAFT: MY MINDFUL WORK OF ART**  Help students appreciate the individual discipline, the commitment, and the energy put into this 23-week process.  Talk about and reflect on the experience. Clarify any remaining issues or concerns about applying CRAFT to their Higher Music Studies or daily life. Reflect on what each student found useful and will carry forward.  Make the students reflect on their own contribution to themselves and the world.    Guide the students through the practice of the Creative Meditation: “From that day until now”.  Make the students reflect on all the personal work they did and be aware of all their changes through the practice: **“My mindful craft work here and from now on”** | **CRAFT: MY HEARTFUL WORK OF ART**  Help students appreciate the individual discipline, the commitment, and the energy put into this 23-week process.  Talk about and reflect on the experience. Clarify any remaining issues or concerns about applying CRAFT to their Higher Music Studies or daily life. Reflect on what each student found useful and will carry forward.  Make the students reflect on their own contribution to themselves and the world.    Guide the students through the practice of the Creative Meditation: “From that day until now”.  Make the students reflect on all the personal work they did and be aware of all their changes through the practice: **“My heartful craft work here and from now on”** |

ADAPTATION OF THE PROGRAM TO THE COVID-19 SITUATION

Due to the pandemic situation, the contents from session 17^th^ to 23^rd^ were taught online and all the music creative practices and live music meditation practices belonging to both subjects were not imparted. In order to offer more tools to deal with the unprecedented stressful situation, compassion and more *metta*-meditations, which usually belong to the emotional intelligence subject, except *metta* towards themselves that is taught in mindfulness, were also worked and practice with the mindfulness group to foster self-care, empathy and to promote more heartfelt responses to the situation. Also, the practice of yoga *nidra*, that it is currently used only with the mindfulness group, was offered to the emotional intelligence group, because of its possible contribution to restore wellbeing and increase relaxation. Specific meditations dealing with uncertainty and a special chapter on time management adapted to the lockdown teaching-learning situation were included in both groups as well.

COMPETENCES

The 5 elements of the CRAFT program are also connected to the 5 emotional competences described by Bizquerra and Pérez (2007): Emotional consciousness, emotional regulation, emotional autonomy, social competence and competences for life and wellbeing. The CRAFT program aims specifically to develop 13 of the 16 transversal competences (CT1, CT2, CT3, CT6, CT7, CT8, CT9, CT10, CT11, CT12, CT13, CT15, CT16) registered on the **Royal Decree law 631/2010 and decree-law 260/2011** published in the BOJA (Boletín Oficial de la Junta de Andalucía) for higher education music studies, as shown in the teaching guides of both subjects: emotional intelligence and mindfulness.

**Royal Decree law 631/2010 and decree-law 260/2011**

CT1- Organize and plan the work in an efficient and motivational way.

CT2-Collect significant information and be able to analyze it, synthesize it, and manage it properly.

CT3- Solve problems and make decisions responding to the objectives of the performing work

CT4- Efficient use of information and communication technologies.
CT6- Be self-critic about their own and interpersonal professional performance.
CT7- Apply communicative skills and constructive criticism in teamwork.

CT8- Develop reasonably and critically ideas and argumentations.

CT9- Be able to integrate themselves properly in multidisciplinary teams and in a diversity of cultural contexts.

CT10- Lead and manage work teams.

CT 11- Develop in the working practice a professional ethics based on appreciation and aesthetic sensitivity, on environmental concerns, and towards diversity.

CT12- Adapt themselves under competitive conditions to the cultural, social and artistic changes that occur in the professional environment and select the appropriate pathways for continuous training.

CT13- Search for excellence and quality in their professional activity

CT 15- Work autonomously and value the importance of the initiative and entrepreneurial spirit in the professional work.

CT16-Use the means and resources available with responsibility towards the cultural and environmental patrimony.

**COMPETENCIAS TRANSVERSALES (1)**

CT1.-Organizar y planificar el trabajo de forma eficiente y motivadora.

CT2.-Recoger información significativa, analizarla, sintetizarla y gestionarla adecuadamente.

CT3-Solucionar problemas y tomar decisiones que respondan a los objetivos del trabajo que se realiza

CT4. Utilizar eficientemente las tecnologías de la información y la comunicación

CT5. Comprender y utilizar al menos una lengua extranjera en el ámbito de su desarrollo profesional

CT6.-Realizar autocrítica hacia el propio desempeño profesional e interpersonal.

CT7. Utilizar las habilidades comunicativas y la crítica constructiva en el trabajo en equipo.

CT8.-Desarrollar razonada y críticamente ideas y argumentos.

CT9.-Integrarse adecuadamente en equipos multidisciplinares y en contextos culturales diversos.

CT10-Liderar y gestionar grupos de trabajo

CT11.-Desarrollar en la práctica laboral una ética profesional basada en el apreciación y sensibilidad estética, medioambiental y hacia la diversidad.

CT12.-Adaptarse, en condiciones de competitividad a los cambios culturales, sociales y artísticos ya los avances que se producen en el ámbito profesional y seleccionar los cauces adecuados de formación continuada.

CT13.-Buscar la excelencia y la calidad en su actividad profesional.

CT14-Dominar la metodología de investigación en la generación de proyectos, ideas y soluciones viables.

CT15.-Trabajar de forma autónoma y valorar la importancia de la iniciativa y el espíritu emprendedor en el ejercicio profesional.

CT16.-Usar los medios y recursos a su alcance con responsabilidad hacia el patrimonio cultural y medioambiental
